# Novel Roles of Chloroquine and Hydroxychloroquine in Graves’ Orbitopathy Therapy by Targeting Orbital Fibroblasts

**DOI:** 10.1210/clinem/dgaa161

**Published:** 2020-03-31

**Authors:** Yan Guo, Hai Li, Xueying Chen, Huasheng Yang, Hongyu Guan, Xiaoying He, Yuxin Chen, Sunil Pokharel, Haipeng Xiao, Yanbing Li

**Affiliations:** 1 Department of Endocrinology and Diabetes Center, The First Aﬃliated Hospital, Sun Yat-sen University, Guangzhou, China; 2 State Key Laboratory of Ophthalmology, Zhongshan Ophthalmic Center, Sun Yat-sen University, Guangzhou, China

**Keywords:** Graves’ orbitopathy, chloroquine, hydroxychloroquine, autophagy, adipogenesis, orbital fibroblasts

## Abstract

**Context:**

Graves’ orbitopathy (GO) causes infiltrative exophthalmos by inducing excessive proliferation, adipogenesis, and glycosaminoglycan production in orbital fibroblasts (OFs). Interference with OF autophagy is a potential therapy for proptosis.

**Objectives:**

Here, we aimed to evaluate the effects of chloroquine (CQ) and hydroxychloroquine (HCQ), the autophagy inhibitors commonly used in clinical practice, on OFs.

**Design/Setting/Participants:**

OFs isolated from patients with GO (GO-OFs) or control individuals (non-GO-OFs) were cultured in proliferation medium (PM) or subjected to differentiation medium. OFs were treated with CQ or HCQ (0, 0.5, 2, and 10 μM), and subsequently examined in vitro.

**Main Outcome Measures:**

CCK-8, EdU incorporation, and flow cytometry assays were used to assess cellular viability. Adipogenesis was assessed with Western blot analysis, real-time polymerase chain reaction (PCR) , and Oil Red O staining. Hyaluronan production was determined by real-time PCR and enzyme-linked immunosorbent assay. Autophagy flux was detected through red fluorescent protein (RFP)-green fluorescent protein (GFP)-LC3 fluorescence staining and Western blot analyses.

**Results:**

CQ/HCQ halted proliferation and adipogenesis in GO-OFs in a concentration-dependent manner through blockage of autophagy, phenotypes that were not detected in non-GO-OFs. The inhibitory effect of CQ/HCQ on hyaluronan secretion of GO-OFs was also concentration dependent, mediated by downregulation of hyaluronan synthase 2 rather than hyaluronidases. Moreover, CQ (10 μM) induced GO-OF apoptosis without aggravating oxidative stress.

**Conclusions:**

The antimalarials CQ/HCQ affect proliferation, adipogenesis, and hyaluronan generation in GO-OFs by inhibiting autophagy, providing evidence that they can be used to treat GO as autophagy inhibitors.

Graves’ orbitopathy (GO) is a common extrathyroidal manifestation of Graves’ disease (1). The major clinical manifestation of GO is proptosis, which negatively impacts patients’ psychosocial wellbeing, and even damages eyesight in 3% to 5% of cases (2,3). Glucocorticoids (GCs), alone or in combination with radiotherapy, has become the mainstay therapy for moderate-to-severe active GO (4, 5). However, GCs can cause dose-limiting adverse reactions (4). In addition, patients that are GC refractory/dependent or have inactive GO benefit little from GCs (6). Persistent exophthalmos and cosmetic concerns at the stable phase of GO can be remedied with surgery (7). It remains an intractable condition for which it is urgent to discover new treatments for GO.

In addition, a series of more specific biologicals targeting cytokines and immunocytes have been considered for active GO, such as rituximab (RTX), which targets against the B-lymphocyte antigen CD20 (8-12). However, despite the favorable anti-inflammatory effects of these treatments, they were not found to be effective on proptosis, especially at the inactive stage (13,14). At the active stage, orbital fibroblasts (OFs) exhibit robust proliferation and adipogenesis, and secrete excessive inflammatory mediators and hydrophilic glycosaminoglycans, specifically hyaluronan (HA). OFs play a central role as the effectors in the pathology of tissue remodeling (15). Tissue remodeling is predominantly caused by chronic fibrosis at the inactive stage when HA deposition and adipogenesis may persist (16). In this context, OFs are good potential targets for novel therapies. Monoclonal antibodies and biologicals targeting the surface receptors of OFs have emerged. The small molecular antagonists and a blocking antibody targeting thyroid stimulating hormone receptor (TSHR) were proved to be effective in vitro and animal models (17-20). But they have yet to be used in clinical settings. A phase III trial demonstrated that teprotumumab ameliorates proptosis and the inflammation state in active GO patients by targeting insulin-like growth factor-1R (6). Nonetheless, the effect of teprotumumab on proptosis in patients with inactive GO has not been determined. Collectively, there is unmet need for new therapies targeting OFs (21).

Autophagy has recently been reported to participate in the pathogenesis of GO, which makes it a potential therapeutic target (22). Blocking autophagy flux with the autophagy inhibitor bafilomycin A1 or knocking down Atg5 can reduce adipogenesis in OFs (22). Icariin and astragaloside IV have been suggested to inhibit adipogenic differentiation of OFs by inhibiting autophagy (23,24). Nevertheless, these autophagy inhibitors have a long way to go before clinical translation.

Chloroquine (CQ) and its derivative hydroxychloroquine (HCQ) prevent degradation of autophagic cargo by blocking acidification of the lysosomal compartment (25). They are autophagy inhibitors with the most potential for clinical application, as they are well-established drugs approved by the Food and Drug Administration. However, whether CQ and HCQ exert therapeutic effects on OFs isolated from patients with GO (GO-OFs) has not been determined. Here, we examined the potential effects of CQ and HCQ on cell viability, adipogenesis, and HA production in GO-OFs in vitro.

## Materials and Methods

### Adipose tissue collection and ethics statements

Orbital adipose tissue samples were collected from 10 patients diagnosed with GO, and from 9 non-GO participants during surgery at Zhongshan Ophthalmic Center, Sun Yat-sen University. The 10 patients with GO received no steroid treatments within 3 months prior to their decompression surgery. Seven cases of GO were revealed to be euthyroid during surgery while 3 cases exhibited subclinical hypothyroidism. The clinical characteristics of the participants in the GO group and non-GO group are summarized in Tables 1 and 2, respectively. All tissue specimens were obtained with the approval of the Institutional Human Research Ethics Committee and were performed in accordance with the principles set out in the Declaration of Helsinki. Written informed consent was obtained from each patient.

**Table 1.** Clinical characteristics of patients with GO in the study

Age (mean/range)	45.6/25-73
Gender (M/F)	3/7
Smoking (Y/N)	0/10
GD history (Y/N)	10/0
Treatment for GD	
Antithyroid medicine	4
Radioactive iodine therapy	3
Surgery	3
Duration of GO (range)	3 months – 16years
CAS score (≤3)	
0	4
1	3
2	2
3	1
Proptosis (range, R/L, mm)	8-22/12-23
Previous treatment	
Radiation	1
GCs	6
None	3

Abbreviations: GD, Graves’ disease; GO, Graves’ orbitopathy; CAS, clinical activity score; GC, glucocorticoid; F, female; M, male; Y, yes; N, no; R, right; L, left.

**Table 2.** Clinical characteristics of patients without GO in the study

Age (mean/range)	42.6/20-64
Gender (M/F)	4/5
Smoking (Y/N)	0/9
GD history (Y/N)	1/8
GO history (Y/N)	0/9
Diagnosis	
Orbital neurilemmoma	1
Choroidal malignant melanoma	5
Exophthalmos	1
Orbital cavernous hemangioma	2
Surgery	
Orbital neoplasm excision	3
Ophthalmectomy	5
Orbital adipectomy	1

Abbreviations: GD, Graves’ disease; GO, Graves’ orbitopathy; F, female; M, male; Y, yes; N, no.

### OF isolation and culture

Orbital adipose tissues were immediately rinsed in phosphate-buffered saline (PBS) (Gibco Laboratories, New York, USA) supplemented with 200 IU/mL penicillin and 200 mg/mL streptomycin. The tissues were then washed twice with PBS, minced with scissors, and digested with 1 mg/mL collagenase type I (MP Biomedicals, California, USA) at 37°C. Digestion was ended by adding Dulbecco’s modified Eagle’s medium nutrient mixture F-12 (Ham) (DMEM-F12) (Gibco Laboratories, New York, USA) supplemented with 10% (vol/vol) fetal bovine serum (FBS) (Gibco Laboratories, New York, USA) after 30 to 60 minutes. The supernatant containing OFs was filtered through a Falcon 40-μm nylon cell strainer (Corning, New York, USA). After centrifugation, the OFs contained in the precipitate were cultivated with proliferation medium (PM) (DMEM-F12 containing 10% FBS, 100 IU/mL penicillin and 100 mg/mL streptomycin) in the 9-cm disks at 37°C in a 5% CO_2_ humidiﬁed incubator. The red blood cells were removed through exchange of PM. When the OFs reached 80% to 90% conﬂuence, they were digested with 0.25% ethylenediamine tetra-acetate-free trypsin (Solarbio, Beijing, China) and passaged. Cells in passages 2 to 6 were used in subsequent assays, for which not all samples were included in each experiment. Each experiment was repeated using OFs from at least 3 independent specimens. OFs from both sexes were used without preference.

### Adipogenic differentiation

To induce adipogenic differentiation, PM was replaced with a commercial adipogenic differentiation medium (DM) (SALIAI, Guangzhou, China) supplemented with or without CQ (Sigma-Aldrich, Missouri, USA)/HCQ (TCI, Shanghai, China) after cells reached confluence. The medium was replaced with fresh DM every 2 to 3 days according to the instruction manual. Ten days later, differentiated GO-OFs were stained with Oil Red O or lysed to conduct subsequent Western blot analysis. To detect the messenger ribonucleic acid (mRNA) expression profiles of adipogenic markers, OFs were lysed after 4 days of differentiation.

### Oil Red O staining

The differentiated OFs were gently washed with PBS and fixed in 4% paraformaldehyde at room temperature for 30 minutes. After being washed with distilled water, the fixed OFs were stained with Oil Red O working solution from the DM kit (SALIAI, Guangzhou, China) for 1 hour. The cells were observed and photographed using a microscope (Olympus IX71, Tokyo, Japan). To quantify lipid accumulation, isopropanol was added to the stained OFs. The optical density (OD) of each well was measured at 450 nm using a SpectraMax i3x microplate reader (Molecular Devices, CA, USA).

### Cell proliferation assays

Cellular viability was assessed using a Cell Counting Kit-8 (CCK-8) assay kit. At the indicated time point, CCK-8 reagent diluted at a 10:1 ratio was added to the medium, and the cells were cultured for another 4 hours before the OD value of each well was measured at 450 nm using a SpectraMax i3x microplate reader (Molecular Devices, California, USA). DNA synthesis was determined by 5-ethynyl-2′-deoxyuridine (EdU) incorporation assay using a Cell Light EdU DNA Imaging Kit (RiboBio Co., Guangzhou, China), as described in our previous study (26,27). Images were obtained and analyzed by a Lionheart FX Automated Live Cell Imager (BioTex, Winooski, USA). A Cell Cycle Detection Kit (KeyGEN BioTECH, Jiangsu, China) was used according to the protocol to assess the cell cycle procession of OFs. A flow cytometer (Beckman Coulter, Florida, USA) was used to measure the DNA content of each sample, and the data were analyzed using the cell cycle analysis software ModFit LT 5.0 (Verity Software House, Maine, USA).

### Apoptosis assays

Annexin V (AV)–propidium iodide (PI) staining was performed using an AV-PI apoptosis detection kit (Vazyme, Nanjing, China), according to the product instructions. Stained cells were detected by a CytoFLEX flow cytometer (Beckman Coulter, Florida, USA) and the data were analyzed using FlowJo Software (Becton, Dickinson & Company, New Jersey, USA).

### Reactive oxygen species measurement

Reactive oxygen species generation was determined using the fluorescent probe 2′,7′-dichlorodihydrofluorescein (Beyotime, Jiangsu, China). OFs were seeded at a density of 2.5 × 10^5^ cells per well in 6-well plates (Nest, Jiangsu, China), and allowed to grow to 70% to 80% confluence before being treated with CQ (10 μM) (Sigma-Aldrich, Missouri, USA) for 72 hours. The indicated cells were trypsinized and washed with PBS, and incubated in serum-free DMEM-F12 with 2′,7′-dichlorodihydrofluorescein (10 μM) at 37°C in darkness for 30 minutes. Subsequently, the cells were washed with PBS again, and analyzed in a CytoFLEX flow cytometer (Beckman Coulter, Florida, USA). The data were collected using FlowJo Software (Becton, Dickinson & Company, New Jersey, USA).

### Western blot analysis

Western blot analysis was performed according to a standard method, as described previously (26). The antibodies for immunoblotting were used as follows: antiglyceraldehyde phosphate dehydrogenase (GAPDH), antiperilipin-1, antiperoxisome proliferator-activated receptor gamma (PPARγ), antifatty acid binding protein 4 (FABP4), anti-CCAAT enhancer-binding protein alpha (c/EBPα), anti-c/EBPβ, anti-LC3B, and anti-p62 (Cell Signaling, Beverly, MA). The intensity of each band was calculated with ImageJ software (National Institutes of Health, Bethesda, Maryland) and normalized to that of GAPDH.

### RNA extraction, reverse transcription polymerase chain reaction and real-time polymerase chain reaction

Total RNA was extracted from OFs with TRIzol Reagent (Life Technologies, California, USA) as previously described (27). The one-step reverse transcription polymerase chain reaction (RT-PCR) reaction was performed using an RT-PCR kit (Promega, Madison, USA), and real-time PCR was conducted using a CFX384 real-time system or a CFX96 real-time system (Bio-Rad, California, USA), as described in a previous study (27). The primers used for real-time PCR were as follows: perilipin-1 (forward), 5′-ATGAGGACCAGACAGACA-3′; and (reverse), 5′-TCACTGAACTTGTTCTCCT-3′; PPARγ (forward), 5′-TTGCAGTGGGGATGTCTCAT-3′; and (reverse), 5′-TTTC CTGTCAAGATCGCCCT-3′; FABP4 (forward), 5′-AGCACC ATAACCTTAGAT-3′; and (reverse), 5′-CACCACCAGTTTATC ATC-3′; c/EBPα (forward), 5′-TGGACAAGAACAGCAACGAGTA-3′; and (reverse), 5′-ATTGTCACTGGTCAGCTCCAG-3′; c/EBPβ (forward), 5′-GGCTTGTTGCTGTTGATG-3′; and (reverse), 5′-AGGCTTTGTAACCATTCTCA-3′; HAS2 (forward), 5′-CTGGGACGAAGTGTGGATTATGTA-3′; and (reverse), 5′-ACCCGGTTCGTGAGATGC-3′; HYAL1 (forward), 5′-GG AAGTCACAGATGTATG-3′; and (reverse), 5′-TTGTCGTGT CATAGAAGA-3′; HYAL2 (forward), 5′-GACCTGAATGCCT TTGAT-3′; and (reverse), 5′-GCGGTAGAAGATGGTAAT-3′; HYAL3 (forward), 5′-CTGCCACTCAATGCTCTG-3′; and (reverse), 5′ -GCCGAGTTGGTTCTTGTA-3′; GAPDH (forward), 5′-TTGAGGTCAATGAAGGGGTC-3′; and (reverse), 5′-GAAGGTGAAGGTCGGAGTCA-3′.

### Measurement of HA

For measurement of HA, 2 × 10^3^ OFs were seeded in a 96-well plate (Nest, Jiangsu, China) with 200 μL of PM. After the OFs were attached to the plate, PM was replaced with serum-free DMEM-F12. The cells were incubated and then recovered with DMEM-F12 containing 1% FBS with or without human interleukin (IL)-1β (1 ng/mL) (Cell Signaling, Beverly, MA). At the same time, they were treated with or without CQ/HCQ at the indicated concentrations. Forty-eight hours later, the culture supernatant from each group was obtained after centrifugation at 4°C, and HA was measured with an enzyme-linked immunosorbent assay (ELISA) kit (Echelon Bioscience Inc., Salt Lake City, USA) according to the manufacturer’s instructions.

### Fluorescence microscopy

OFs were inoculated into 24-well plates (Nest, Jiangsu, China) with PM at a density of 1 × 10^5^ cells/well with PM. Once the OFs reached 70% conﬂuence, lentiviruses expressing RFP-GFP-LC3 plasmid (Hanbio, Shanghai, China) were added to the wells at a multiplicity of infection of 100. The medium was exchanged after 24 hours of infection. After another 24 hours of incubation in the PM, RFP-GFP-LC3-labeled OFs were kept in the PM or stimulated with DM after 48 hours until confluence. Fluorescent images of each group were respectively obtained at the indicated times (days 0, 1, and 2) with a fluorescence microscope (Olympus IX71, Tokyo, Japan). The LC3 puncta were counted using the ImageJ software (National Institutes of Health, Bethesda, Maryland) to confirm the induction of autophagy.

### Statistical analysis

Each experiment was performed using OFs isolated from 3 to 6 patients. The data are presented as the mean ± standard error of the mean (SEM) and were analyzed by applying independent-samples t tests, nonparametric tests (Mann–Whitney U 1ests) or 1-way analysis of variance (least significant difference [LSD]) using SPSS 20.0 software (SPSS Inc., Illinois, USA). Statistical significance required *P *< .05.

## Results

### CQ and HCQ blocked autophagy flux of GO-OFs at the noncytotoxic concentrations.

**Figure 1.**
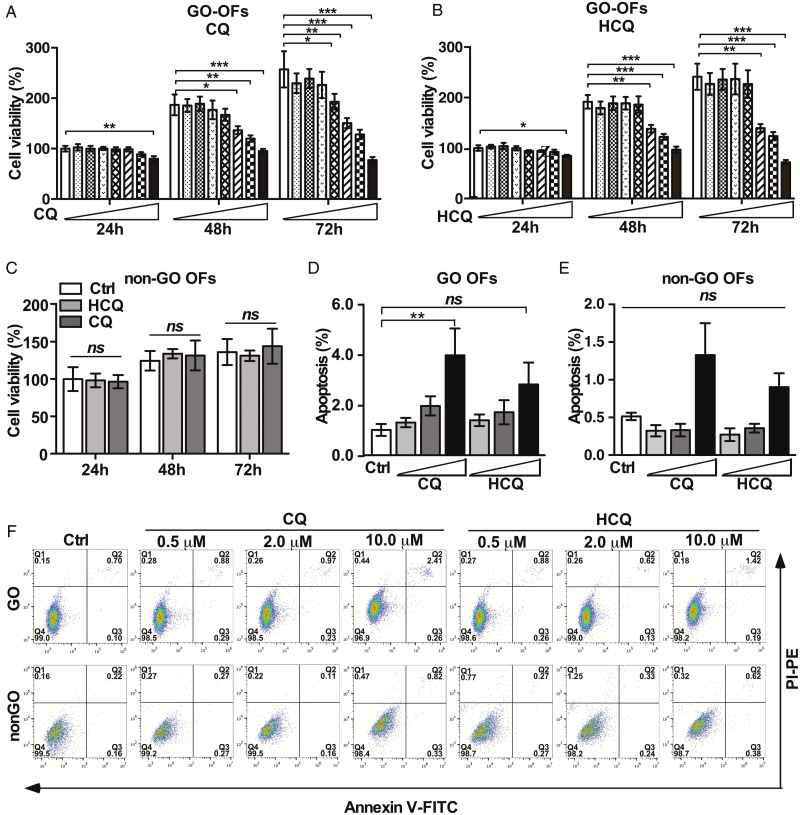
Effect of CQ and HCQ on the cellular viability of OFs from GO and non-GO cases. (A) OFs obtained from 6 GO patients were treated with increasing concentrations of CQ (0, 0.5, 1, 5, 10, 25, 50, and 100 μM) in PM for 24, 48, and 72 hours, respectively. Cell viability is presented as the percentage relative to the viability of the untreated cells. (B) OFs obtained from 6 GO patients were treated with increasing concentrations of HCQ (0, 0.5, 1, 5, 10, 25, 50, and 100 μM) in PM for 24, 48, and 72 hours, respectively. Cell viability is presented as the percentage relative to the viability of the untreated cells. (C) OFs obtained from 6 non-GO patients were treated with or without CQ and HCQ (10 μM) in PM for 24, 48, and 72 hours, respectively. Cell viability is presented as the percentage relative to the viability of the untreated cells. (D) Quantiﬁcation of cell apoptosis in each GO group (Ctrl, CQ [0.5, 2,10 μM], and HCQ [0.5, 2,10 μM]) as detected by ﬂow cytometry using AV-FITC/PI staining, n = 5. (E) Quantiﬁcation about cell apoptosis in each non-GO group (Ctrl, CQ [0.5, 2,10 μM], HCQ [0.5, 2,10 μM]) as detected by ﬂow cytometry using AV-FITC/PI staining, n = 5. (F) Experimental diagrams of cell apoptosis induced by CQ/HCQ at the indicated concentration in GO-OFs and non-GO-OFs by ﬂow cytometry using AV-FITC/PI staining. For (A), (B), (C), (D) and (E), the bar graph data are shown as the mean ± standard error of the mean. *P < .05, ^**^P < .01, ^***^P < .001, ^*ns*^*P* ≥ .05 versus Ctrl group.

To explore the noncytotoxic concentrations of CQ and HCQ in OFs, CCK-8 assays were performed. As demonstrated in Fig. 1A and 1B, CQ and HCQ showed low cytotoxicity to OFs at concentrations ≤10 μM. In addition, neither CQ nor HCQ at 10 μM caused cytotoxicity in non-GO-OFs, as shown in Fig. 1C. The cell viability of GO-OFs treated with 10 μM CQ for 72 h decreased to 76.78 ± 4.89% (n = 6) of that observed in the control (Ctrl) level, whereas this effect was not observed for HCQ, indicating that HCQ has a lower toxicity than CQ. To determine whether the decrease in cell viability was due to apoptosis or lower proliferation, we further treated GO-OFs and non-GO-OFs with CQ/HCQ (0, 0.5, 2, and 10 μM) for up to 72 hours before analyzing apoptosis. Above all, the basal apoptosis in GO-OFs was higher than that in non-GO-OFs (Fig. 1D-F). CQ/HCQ exerted no obvious proapoptotic effect on GO- and non-GO-OFs, except for CQ at 10 μM (Ctrl 0.80 ± 0.53% vs CQ 2.67 ± 2.39% in GO-OFs, *P* = .002). But we noticed that the apoptosis rate in GO-OFs induced by CQ was relatively low, without causing higher reactive oxygen species levels (all supplementary material and figures are presented in a digital research materials repository (28)). Thus we speculated that antiproliferative effects may also have been involved. Western blot analysis results confirmed that p62 and total LC3 abundance in GO-OFs were upregulated by CQ and HCQ in a concentration-dependent manner (see (28)). Collectively, these findings indicated that CQ and HCQ at the tested concentrations (0.5, 2, 10 μM) were suitable for the subsequent experiments.

### Inhibitory effects of CQ and HCQ on the cellular proliferation of OFs

**Figure 2.**
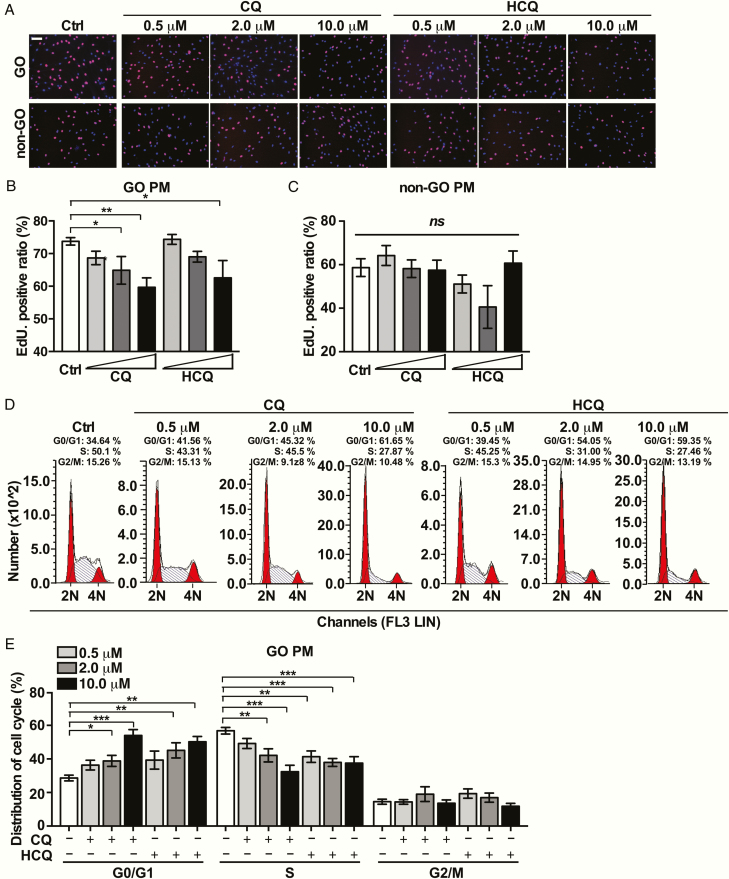
Effects of CQ and HCQ on the cellular proliferation of GO- and non-GO-OFs in PM. (A) Representative images of the EdU incorporation assay results in GO-OFs and non-GO-OFs treated with CQ/HCQ at the indicated concentrations in PM. The cells were observed using a fluorescence microscope, scale bars = 100 μm. Red: EdU, Blue: 4′,6-diamidino-2-phenylindole (DAPI). (B) Quantiﬁcation of the EdU incorporation assay results of GO-OFs (Ctrl, CQ (0.5, 2,10 μM], HCQ [0.5, 2,10 μM]), n = 5. (C) Quantiﬁcation of the EdU incorporation assay results of non-GO-OFs (Ctrl, CQ [0.5, 2,10 μM], HCQ [0.5, 2,10 μM]), n = 5. (D) Diagrams of the cell cycle of each group of GO cases in PM, as determined by flow cytometry. (E) Cartogram of cell cycle distribution of each GO-OFs group in PM, n = 5. For (B), (C), and (E), the summarized data are reported as the mean ± standard error of the mean. *P < .05, ^**^P < .01, ^***^P < .001, ^*ns*^*P* ≥ .05 versus the Ctrl group.

To further evaluate the effects of CQ and HCQ on OF proliferation, we conducted EdU assays and flow cytometry analyses on OFs in PM. The basal cellular proliferation rate of in GO-OFs was also higher than that in non-GO-OFs (Fig. 2A-C). CQ and HCQ significantly lowered the EdU-positive ratio of GO-OFs in a concentration-dependent manner (HCQ vs Ctrl: 10 μM [*P* = .016]; CQ vs Ctrl: 2 μM [*P* = .041], 10 μM [*P* = .003]). No significant difference was observed in the EdU ratio among groups of non-GO-OFs (HCQ vs Ctrl: 10 μM [*P* = .222]; CQ vs Ctrl: 10 μM [*P* = .056]), as shown in Fig. 2A and 2C. The proportion of cells in G0/G1 phase was upregulated in the CQ and HCQ-treated groups in a concentration-dependent way, as presented in Fig. 2D and 2E. As a result, CQ and HCQ prevented excessive expansion of orbital preadipocytes.

CQ and HCQ suppress the mitotic clonal expansion (MCE) of GO-OFs at the early stage of differentiation

**Figure 3.**
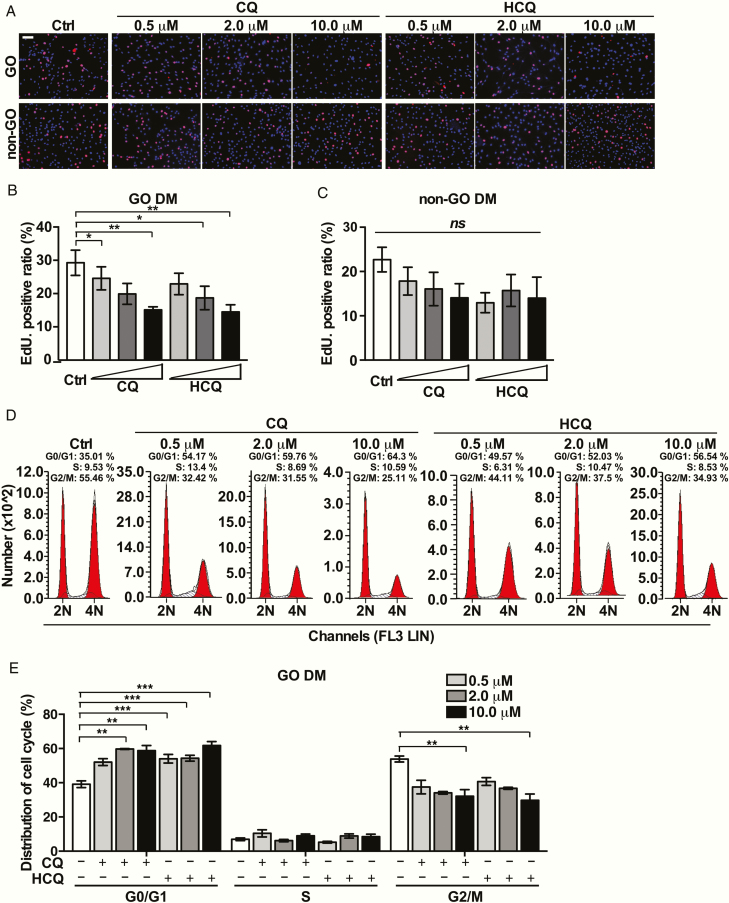
Effects of CQ and HCQ on the cellular proliferation of GO/non-GO-OFs in DM. Forty-eight hours after GO-OFs were arrested, they were stimulated by DM with or without CQ/HCQ (0.5, 2, 10 μM) before analysis. (A) Representative images of the EdU incorporation assay results for each group of GO-OFs and non-GO-OFs treated with CQ/HCQ at the indicated concentrations in DM, scale bars = 100 μm. Red: EdU, Blue: DAPI. (B) Quantiﬁcation of the EdU incorporation assay results for GO-OFs (Ctrl, CQ [0.5, 2,10 μM], HCQ [0.5, 2,10 μM]), n = 5. (C) Quantiﬁcation of the EdU incorporation assay results for non-GO-OFs (Ctrl, CQ [0.5, 2,10 μM], and HCQ [0.5, 2,10 μM]), n = 5. (D) Experimental diagrams of the cell cycle distribution for each group of GO-OFs in DM, as determined by flow cytometry. (E) Cartogram about cell cycle distribution for each group of GO-OFs in DM, n = 5. For (B), (C) and (E), the data are presented as the mean ± SEM. *P < .05, ^**^P < .01, ^***^P < .001, ^*ns*^*P* ≥ .05 versus the Ctrl group.

We determined that the confluent GO-OFs reenter the cell cycle under stimulation of DM after contact inhibition (see (28)). The basal cellular proliferation of GO-OFs was also higher than that observed for non-GO-OFs in DM (Fig. 3A-C). CQ/HCQ dose dependently prevented GO-OFs from regaining DNA synthesis ability after growth arrest (Fig. 3A and 3B). We detected that CQ or HCQ slightly decreased the EdU-positive ratio in the non-GO-OFs, but not significantly (HCQ vs Ctrl: 10 μM [*P* = .096]; CQ vs Ctrl: 10 μM [*P* = .078]), as represented in Fig. 3A and 3C. Consistently, CQ or HCQ each increased the proportion of OFs in G0/G1 phase in DM in a concentration-dependent manner (Fig. 3D and 3E). In order to further exclude the possibility that CQ and HCQ blocked the MCE of OFs by inducing cytotoxicity, we conducted a CCK-8 assay on GO-OFs and non-GO-OFs under the same conditions used for cell cycle detection (see (28)).

### CQ and HCQ exerted antiadipogenic effects on GO-OFs

**Figure 4.**
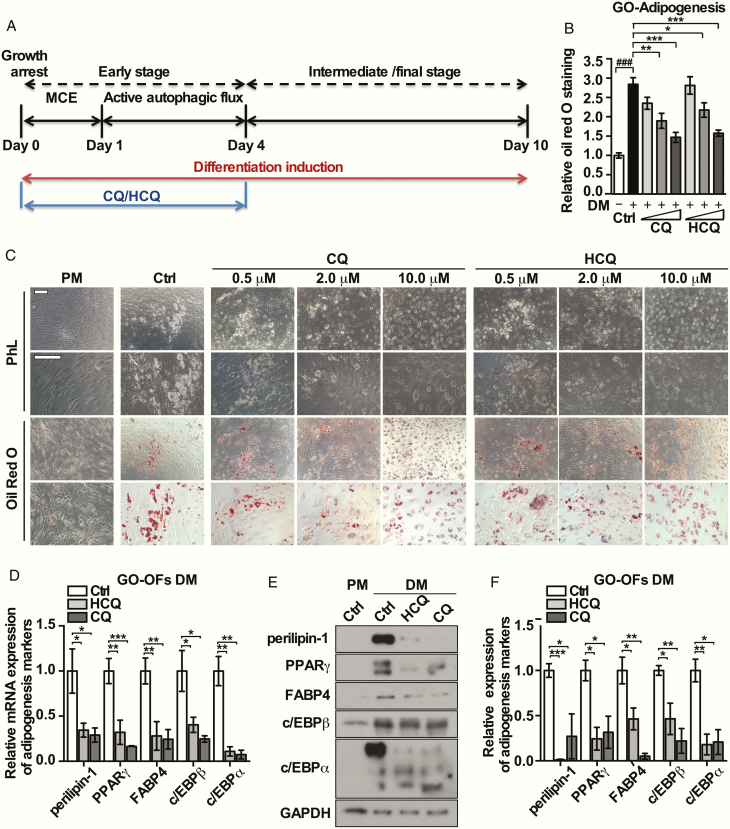
Effects of CQ and HCQ on adipogenesis in GO-OFs in vitro. (A) Forty-eight hours after growth was arrested in confluent GO-OFs, adipogenesis was stimulated with DM supplemented with or without CQ/HCQ (0.5, 2, 10 μM) for the indicated time periods. (B) Microscopic detection was performed to assess the characteristic morphological changes of adipogenesis associated with Oil Red O staining for each group of GO cases (PM-Ctrl, DM-Ctrl, DM-CQ [0.5, 2,10 μM] DM-HCQ [0.5, 2,10 μM]), scale bars = 200 μm. (C) Relative quantiﬁcation of the Oil Red O staining for each group of GO-OFs, via detection of the OD values at 450 nm after stained cells were solubilized, n = 5. (D) After 4 days of adipogenic induction with or without CQ/HCQ (10 μM), the mRNA levels of the adipogenic markers c/EBPα/β, PPARγ, perilipin-1 and FABP4, were determined by real-time PCR, n = 5. (E) After 10 days of adipogenesis with or without CQ/HCQ (10 μM), the protein expression of the indicated adipogenesis markers in each group was assessed by Western blot analysis. (F) The protein levels were quantified, normalized to the level of GAPDH for each sample, and analyzed, n = 3. For (C), (D), and (F), the results which were derived from 3 to 5 independent GO-OF samples and are expressed as the mean ± standard error of the mean. *P < .05, ^**^P < .01. ^***^P < .001 versus the Ctrl group; ^###^*P* < .001 versus the PM Ctrl group.

We confirmed that the transformation of LC3-I to LC3-II, represented as the LC3-II/I ratio, and the numbers of autophagosomes in GO-OFs were markedly increased by an adipogenic cocktail at the early stage (28). CQ and HCQ were administered separately during the first 4 days of differentiation, as shown in Fig. 4A. Typical morphological changes such as cell rounding and accumulation of large lipid droplets were observed in the GO-OFs after induction for 10 days; these changes were different from those in the groups treated by CQ/HCQ (2 and 10 μM) (Fig. 4B). Oil Red O staining calculated as the OD value, was significantly lower by CQ/HCQ at the indicated concentrations (CQ vs Ctrl: 2 μM [*P* = .002], 10 μM [*P* = .000068]; HCQ vs Ctrl: 2 μM [*P* = .016], 10 μM [*P* = .0001]) (Fig. 4C). Furthermore, we performed real-time PCR and Western blot analysis to detect adipogenic markers. As shown in Fig. 4D-F, CQ and HCQ (10 μM) were able to dampen the expressions of perilipin-1, PPARγ, FABP4, and c/EBPα/β at both the mRNA and protein levels. Oil Red O staining showed that lipid droplets were increased in non-GO-OFs, but to a lesser extent than that observed for GO-OFs, and these increases were not significantly attenuated by CQ or HCQ (CQ [10 μM] vs Ctrl: *P* = .173; HCQ [10 μM] vs Ctrl: *P* = .566), as shown in (28). CQ and HCQ (10 μM) added in DM for 10 days did not alter cell viability neither in GO-OFs or non-GO-OFs (as shown in (28)).

### CQ and HCQ attenuated HA production in GO-OFs

**Figure 5.**
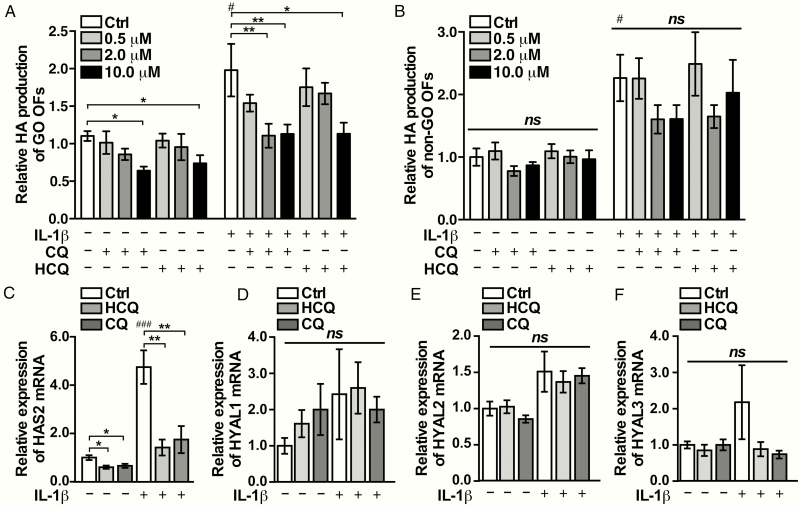
HA production in GO-OFs is downregulated by CQ or HCQ in vitro. GO-OFs cultivated in DMEM-F12 supplemented with 1% FBS, were treated with or without 1 ng/mL of IL-1β together with CQ/HCQ (0, 0.5, 2, 10 μM) for 48 hours. (A) HA secretion in each GO group was determined by ELISA, n = 5. (B) HA secretion of each group of non-GO was determined by ELISA, n = 5. (C) The mRNA levels of HAS2 in the GO cases in each group (Ctrl, CQ 10 μM, HCQ 10 μM), n = 6. (D) mRNA levels of HYAL1 of each group (Ctrl, CQ 10 μM, HCQ 10 μM) in the GO cases were shown, n = 5. (E) mRNA levels of HYAL2 in the GO cases in each group (Ctrl, CQ 10 μM, HCQ 10 μM), n = 6. (F) mRNA levels of HYAL3 in the GO cases in each group (Ctrl, CQ 10 μM, HCQ 10 μM), n = 5. The data are shown as the mean ± SEM, *P < .05, ^**^P < .01, ^*ns*^*P* ≥ .05 versus the Ctrl group; ^#^*P* < .05, ^###^*P* < .001 versus the IL-1β negative Ctrl group.

We found that HA production in GO-OFs with or without stimulation of IL-1β was lowered by CQ and HCQ at the indicated concentrations, which was not the case in non-GO-OFs (Fig. 5A and 5B). Consistent with the HA production detected by ELISA, the mRNA expression of HAS2 was upregulated by IL-1β, and downregulated by CQ and HCQ (10 μM), as shown in Fig. 5C. Similar to the previous studies, we did not detect HYAL4 in the GO-OFs via real-time PCR (data not shown) (29). In addition, the mRNA expression levels of HYAL1, HYAL2, and HYAL3 were not affected by CQ or HCQ in the GO-OFs, as shown in Fig. 5D-F. Neither CQ nor HCQ (10 μM) led to cytotoxicity in GO-OFs or non-GO-OFs under the conditions used in the HA generation experiments (as shown in (28)).

## Discussion

Even though the current treatments for GO are effective in controlling inflammation, there are limited options except surgery available for those who are suffering from exophthalmos and disfigurement. In the current study, we demonstrated CQ and HCQ decrease proliferation, adipogenesis, and HA generation in GO-OFs. As demonstrated by Kuriyan et al., there are 2 types of OFs in the orbit of GO. Type II OFs favor rapid proliferation, whereas type I OFs tend to undergo adipogenesis (30). Here, CQ and HCQ exert both the cytostatic and antiadipogenic effects, indicating that CQ and HCQ may be suitable treatment for different clinical subtypes of GO patients. Extraocular muscle enlargement and orbital fat expansion can be caused by HA deposition (31). The inhibitory effects of CQ and HCQ on HA production that we observed in the current study provide additional evidence that patients of different subtypes may respond well to the treatment of CQ and HCQ. Albeit fibrosis is the predominant mechanism of tissue remodeling at the late phase of GO, adipogenesis and HA generation can persist in facilitating expansion of orbital contents (16,32). Moreover, it has been illustrated that HA is a driving factor required for fibrosis, suggesting that CQ/HCQ could exert antifibrotic effect on the inactive GO through interference with HA production (33).

The steady blood levels of CQ range between 0.2 and 5 μM in patients with rheumatoid arthritis (34). The administration of HCQ in 171 patients with stable lupus maintained steady blood concentrations in the micromolar range, between 0.3 and 1.9 μM as reported by Costedoat-Chalumeau et al. (35). We applied several concentrations (0.5, 2, 10 μM) at the scale of the physiological concentrations to the in vitro experiments here, which further highlights the practical value of CQ/HCQ for GO treatment. With better tolerance and lower cytotoxicity than CQ, HCQ at maximum oral dosage could achieve a peak blood concentration at 10 μM (25). We found that HCQ at 10 μM affects the pathology of GO-OFs in vitro, without inducing obvious apoptosis, making it safer than CQ in clinical application.

Autophagy is accompanied by rapid alterations of cytosolic compositions, turnover of routine proteins and organelles, as well as modification of cell membrane receptors, nuclear transcriptional factors and cytoskeletal dynamics, suggesting its important role in cell differentiation (36). Skop et al. reported for the first time that the early but not late period of adipogenesis is when inhibition of autophagy has an antiadipogenic effect (37). During early adipogenesis, autophagy ensures the essential components for MCE which is prerequisite for adipogenesis; autophagy is also required for mitochondria degradation which is a vital process in the early stage (37). Here, we confirmed for the first time that GO-OFs also undergo MCE at the early stage of adipogenesis, similar to that observed for other kinds of pre-adipocytes, which could be interrupted by CQ/HCQ. These results indicate that blockage of adipogenesis by CQ/HCQ is mediated by the antiproliferative effect, at least in part. It was also reported that p62 impairs adipocyte differentiation via suppression of basal ERK activity at the early phase (38). To the best of our knowledge, ERK activation is associated with cell cycle progress (39). Interestingly, blockage of autophagy at the early stage of adipogenesis can dampen the MCE process by upregulating p62. In this study, we observed that along with upregulation of p62 induced by CQ/HCQ, adipogenesis was prevented. Notably, we found that when OFs were treated with CQ/HCQ at the intermediate or final stage of adipogenesis, the inhibitory effect disappeared (data not shown), implying that CQ/HCQ interrupts adipogenesis through autophagy inhibition. In addition, autophagy facilitates the degradation of the negative regulators of adipocyte differentiation such as Klf2/3 in 3T3-L1 cells (40). Previous studies on mesangial cells and 3T3-L1 cells have shown that HA secretion occurs concurrently with upregulation of cyclin D3 and c/EBPα at the end of the cell cycle, implying that HA biosynthesis requires cell division (41,42). We could infer that the antiproliferative roles of CQ and HCQ also account for the inhibitory effect on HA production. In general, we have provided further proof for the vital roles of autophagy at the early stage of adipogenesis in GO-OFs, suggesting that it could be a potential target for GO therapy. As drugs approved by Food and Drug Administration, CQ/HCQ have more clinical practicability than other autophagy blockers.

Aside from autophagy inhibition, CQ and HCQ exert multiple anti-inflammatory effects (43,44), which include inhibiting chemotaxis and antigen presentation via interference with endosome acidification (45); restricting cytokine production (46); blocking the effects of prostaglandins (47); disturbing toll-like receptor signaling (48); inhibiting calcium signaling of T- and B-cell receptors (49); and downregulating matrix metalloproteinases (50). Given that GO is an autoimmune disease, the anti-inflammatory roles of CQ/HCQ could provide explanations, at least partially, for why CQ/HCQ have a different impact on OFs for GO versus non-GO patients. Accounting for the effects of OFs in the immune system, therapeutic effects of CQ/HCQ could be more potent in vivo toward GO (51,52).

There are some limitations to the current study. Given that the tissues were obtained from patients whose clinical activity score was ≤3, they could not completely represent the inflammatory state of active GO. In addition, similar to other in vitro studies, we only focused on OFs without considering the crosstalk between immunologically active cells and OFs, or the influence of microenvironment. Despite these existing limitations, this model is still the most widely used one to study OF pathophysiology.

In conclusion, CQ and HCQ hold promise for efficaciously regulating the proliferation, adipogenesis, and HA generation of GO-OFs, which play a central role in orbital tissue remolding, primarily by blocking autophagy.

## Abbreviations

AV: annexin
c/EBPα: anti-CCAAT enhancer-binding protein alpha
CQ: chloroquine
DM: differentiation medium
EdU: 5-ethynyl-2′-deoxyuridine
ELISA: enzyme-linked immunosorbent assay
FABP4: antifatty acid binding protein 4
FBS: fetal bovine serum
GAPDH: glyceraldehyde phosphate dehydrogenase
GC: glucocorticoid
GO: Graves’ orbitopathy
HA: hyaluronan
HAS: hyaluronan synthase
HCQ: hydroxychloroquine
MCE: mitotic clonal expansion
mRNA: messenger ribonucleic acid
OD: optical density
OF: orbital fibroblast
PBS: phosphate-buffered saline
PCR: polymerase chain reaction
PI: propidium iodide
PM: proliferation medium
PPARγ: peroxisome proliferator-activated receptor gamma
RTX,: rituximab

## Additional Information

***Disclosure Summary:*** I certify that neither I nor my co-authors have a conflict of interest as described above that is relevant to the subject matter or materials included in this work.

***Data Availability*:** The datasets generated during and/or analyzed during the current study are not publicly available but are available from the corresponding author on reasonable request.

## References

[1] Bartalena L, Pinchera A, Marcocci C Management of Graves’ ophthalmopathy: reality and perspectives. Endocr Rev. 2000;21(2):168-199. 10782363 10.1210/edrv.21.2.0393

[2] Yeatts RP Quality of life in patients with Graves ophthalmopathy. Trans Am Ophthalmol Soc. 2005;103:368-411. 17057811 PMC1447582

[3] Wiersinga WM, Bartalena L Epidemiology and prevention of Graves’ ophthalmopathy. Thyroid. 2002;12(10):855-860. 12487767 10.1089/105072502761016476

[4] Sisti E, Coco B, Menconi F, et al Intravenous glucocorticoid therapy for Graves’ ophthalmopathy and acute liver damage: an epidemiological study. Eur J Endocrinol. 2015;172(3):269-276. 25661744 10.1530/EJE-14-0712

[5] Tanda ML, Bartalena L Efficacy and safety of orbital radiotherapy for graves’ orbitopathy. J Clin Endocrinol Metab. 2012;97(11):3857-3865. 22962421 10.1210/jc.2012-2758

[6] Smith TJ, Kahaly GJ, Ezra DG, et al Teprotumumab for thyroid-associated ophthalmopathy. N Engl J Med. 2017;376(18):1748-1761. 28467880 10.1056/NEJMoa1614949 PMC5718164

[7] Smith TJ Pathogenesis of Graves’ orbitopathy: a 2010 update. J Endocrinol Invest. 2010;33(6):414-421. 20631493 10.1007/BF03346614 PMC6779038

[8] Salvi M, Vannucchi G, Beck-Peccoz P Potential utility of rituximab for Graves’ orbitopathy. J Clin Endocrinol Metab. 2013;98(11):4291-4299. 24009135 10.1210/jc.2013-1804

[9] Paridaens D, van den Bosch WA, van der Loos TL, Krenning EP, van Hagen PM The effect of etanercept on Graves’ ophthalmopathy: a pilot study. Eye. 2005;19(12):1286-1289. 15550932 10.1038/sj.eye.6701768

[10] Ayabe R, Rootman DB, Hwang CJ, Ben-Artzi A, Goldberg R Adalimumab as steroid-sparing treatment of inflammatory-stage thyroid eye disease. Ophthal Plast Reconstr Surg. 2014;30(5):415-419. 10.1097/IOP.0000000000000211 24978425

[11] Ye X, Bo X, Hu X, et al Efficacy and safety of mycophenolate mofetil in patients with active moderate-to-severe Graves’ orbitopathy. Clin Endocrinol. 2017;86(2):247-255. 10.1111/cen.13170 27484048

[12] Perez-Moreiras JV, Gomez-Reino JJ, Maneiro JR, et al; Tocilizumab in Graves Orbitopathy Study Group. Efficacy of tocilizumab in patients with moderate-to-severe corticosteroid-resistant Graves orbitopathy: a randomized clinical trial. Am J Ophthalmol. 2018;195:181-190. 30081019 10.1016/j.ajo.2018.07.038

[13] Perros P, Weightman DR, Crombie AL, Kendall-Taylor P Azathioprine in the treatment of thyroid-associated ophthalmopathy. Acta Endocrinol. 1990;122(1):8-12. 2305608 10.1530/acta.0.1220008

[14] Prummel MF, Mourits MP, Berghout A, et al Prednisone and cyclosporine in the treatment of severe Graves’ ophthalmopathy. N Engl J Med. 1989;321(20):1353-1359. 2519530 10.1056/NEJM198911163212002

[15] Meyer zu Horste M, Stroher E, Berchner-Pfannschmidt U, et al A novel mechanism involved in the pathogenesis of Graves ophthalmopathy (GO): clathrin is a possible targeting molecule for inhibiting local immune response in the orbit. J Clin Endocrinol Metab. 2011;96(11):E1727-E1736. 21917865 10.1210/jc.2011-1156

[16] Potgieser PW, Wiersinga WM, Regensburg NI, Mourits MP Some studies on the natural history of Graves’ orbitopathy: increase in orbital fat is a rather late phenomenon. Eur J Endocrinol. 2015;173(2):149-153. 26142100 10.1530/EJE-14-1140

[17] van Koppen CJ, de Gooyer ME, Karstens WJ, et al. Mechanism of action of a nanomolar potent, allosteric antagonist of the thyroid-stimulating hormone receptor. Br J Pharmacol. 2012;165(7):2314-2324. 22014107 10.1111/j.1476-5381.2011.01709.x PMC3413865

[18] Neumann S, Nir EA, Eliseeva E, et al. A selective TSH receptor antagonist inhibits stimulation of thyroid function in female mice. Endocrinology. 2014;155(1):310-314. 24169564 10.1210/en.2013-1835 PMC3868809

[19] Marcinkowski P, Hoyer I, Specker E, et al. A new highly thyrotropin receptor-selective small-molecule antagonist with potential for the treatment of Graves’ orbitopathy. Thyroid. 2019;29(1):111-123. 30351237 10.1089/thy.2018.0349

[20] Furmaniak J, Sanders J, Young S, et al. In vivo effects of a human thyroid-stimulating monoclonal autoantibody (M22) and a human thyroid-blocking autoantibody (K1-70). Auto Immun Highlights. 2012;3(1):19-25. 26000124 10.1007/s13317-011-0025-9 PMC4389019

[21] Draman MS, Ludgate M Thyroid eye disease- an update. Expert Rev Ophthalmol. 2016;11(4):273-284.

[22] Yoon JS, Lee HJ, Chae MK, Lee EJ Autophagy is involved in the initiation and progression of Graves’ orbitopathy. Thyroid. 2015;25(4):445-454. 25687157 10.1089/thy.2014.0300

[23] Li H, Zhang Y, Min J, Gao L, Zhang R, Yang Y Astragaloside IV attenuates orbital inflammation in Graves’ orbitopathy through suppression of autophagy. Inflamm Res. 2018;67(2):117-127. 29127443 10.1007/s00011-017-1100-0

[24] Li H, Yuan Y, Zhang Y, Zhang X, Gao L, Xu R Icariin inhibits AMPK-dependent autophagy and adipogenesis in adipocytes in vitro and in a model of Graves’ orbitopathy in vivo. Front Physiol. 2017;8:45. 28243204 10.3389/fphys.2017.00045 PMC5303717

[25] Amaravadi RK, Yu D, Lum JJ, et al Autophagy inhibition enhances therapy-induced apoptosis in a Myc-induced model of lymphoma. J Clin Invest. 2007;117(2):326-336. 17235397 10.1172/JCI28833 PMC1765515

[26] Liang W, Guan H, He X, et al Down-regulation of SOSTDC1 promotes thyroid cancer cell proliferation via regulating cyclin A2 and cyclin E2. Oncotarget. 2015;6(31):31780-31791. 26378658 10.18632/oncotarget.5566 PMC4741639

[27] Guo Y, Li H, Guan H, et al Dermatopontin inhibits papillary thyroid cancer cell proliferation through MYC repression. Mol Cell Endocrinol. 2019;480:122-132. 30391671 10.1016/j.mce.2018.10.021

[28] Guo Y, Li H, Chen X, et al Data from: Novel roles of chloroquine and hydroxychloroquine in Graves’ orbitopathy therapy by targeting orbital fibroblasts. v10, Dryad, Dataset. 2020 10.5061/dryad.5tb2rbp1k. PMC7183395 32249902

[29] Zhang L, Grennan-Jones F, Draman MS, et al Possible targets for nonimmunosuppressive therapy of Graves’ orbitopathy. J Clin Endocrinol Metab. 2014;99(7):E1183-1190. 24758182 10.1210/jc.2013-4182

[30] Kuriyan AE, Woeller CF, O’Loughlin CW, Phipps RP, Feldon SE Orbital fibroblasts from thyroid eye disease patients differ in proliferative and adipogenic responses depending on disease subtype. Invest Ophthalmol Vis Sci. 2013;54(12):7370-7377. 24135759 10.1167/iovs.13-12741 PMC3823547

[31] Bahn RS Current Insights into the pathogenesis of Graves’ ophthalmopathy. Horm Metab Res. 2015;47(10):773-778. 26361262 10.1055/s-0035-1555762

[32] Wiersinga WM, Prummel MF Pathogenesis of Graves’ ophthalmopathy--current understanding. J Clin Endocrinol Metab. 2001;86(2):501-503. 11157999 10.1210/jcem.86.2.7338

[33] Albeiroti S, Soroosh A, de la Motte CA Hyaluronan’s role in fibrosis: a pathogenic factor or a passive player? Biomed Res Int. 2015;2015:790203. 26583132 10.1155/2015/790203 PMC4637089

[34] Augustijns P, Geusens P, Verbeke N Chloroquine levels in blood during chronic treatment of patients with rheumatoid arthritis. Eur J Clin Pharmacol. 1992;42(4):429-433. 1307690 10.1007/BF00280130

[35] Costedoat-Chalumeau N, Amoura Z, et al Low blood concentration of hydroxychloroquine is a marker for and predictor of disease exacerbations in patients with systemic lupus erythematosus. Arthritis Rheum. 2006;54(10):3284-3290. 17009263 10.1002/art.22156

[36] Cecconi F, Levine B The role of autophagy in mammalian development: cell makeover rather than cell death. Dev Cell. 2008;15(3):344-357. 18804433 10.1016/j.devcel.2008.08.012 PMC2688784

[37] Skop V, Cahova M, Dankova H, et al Autophagy inhibition in early but not in later stages prevents 3T3-L1 differentiation: effect on mitochondrial remodeling. Differentiation. 2014;87(5):220-229. 25041706 10.1016/j.diff.2014.06.002

[38] Rodriguez A, Duran A, Selloum M, et al Mature-onset obesity and insulin resistance in mice deficient in the signaling adapter p62. Cell Metabol. 2006;3(3):211-222. 10.1016/j.cmet.2006.01.011 16517408

[39] Lewis TS, Shapiro PS, Ahn NG Signal transduction through MAP kinase cascades. Adv Cancer Res. 1998;74:49-139. 9561267 10.1016/s0065-230x(08)60765-4

[40] Guo L, Huang JX, Liu Y, et al Transactivation of Atg4b by C/EBPbeta promotes autophagy to facilitate adipogenesis. Mol Cell Biol. 2013;33(16):3180-3190. 23754749 10.1128/MCB.00193-13 PMC3753907

[41] Wang A, Hascall VC Hyperglycemia, intracellular hyaluronan synthesis, cyclin D3 and autophagy. Autophagy. 2009;5(6):864-865. 19550149 10.4161/auto.9041

[42] Wang A, de la Motte C, Lauer M, Hascall V Hyaluronan matrices in pathobiological processes. FEBS J. 2011;278(9):1412-1418. 21362136 10.1111/j.1742-4658.2011.08069.x PMC4401461

[43] Achan J, Talisuna AO, Erhart A, et al Quinine, an old anti-malarial drug in a modern world: role in the treatment of malaria. Malaria J. 2011;10(1):144. 10.1186/1475-2875-10-144 PMC3121651 21609473

[44] Nakagawa M, Sugawara K, Goto T, Wakui H, Nunomura W Hydroxychloroquine binding to cytoplasmic domain of Band 3 in human erythrocytes: Novel mechanistic insights into drug structure, efficacy and toxicity. Biochem Biophys Res Commun. 2016;473(4):999-1004. 27049308 10.1016/j.bbrc.2016.04.005

[45] Ohkuma S, Poole B Fluorescence probe measurement of the intralysosomal pH in living cells and the perturbation of pH by various agents. Proc Natl Acad Sci U S A. 1978;75(7):3327-3331. 28524 10.1073/pnas.75.7.3327 PMC392768

[46] Sperber K, Quraishi H, Kalb TH, Panja A, Stecher V, Mayer L Selective regulation of cytokine secretion by hydroxychloroquine: inhibition of interleukin 1 alpha (IL-1-alpha) and IL-6 in human monocytes and T cells. J Rheumatol. 1993;20(5):803-808. 8336306

[47] Loffler BM, Bohn E, Hesse B, Kunze H Effects of antimalarial drugs on phospholipase A and lysophospholipase activities in plasma membrane, mitochondrial, microsomal and cytosolic subcellular fractions of rat liver. Biochim Biophys Acta. 1985;835(3):448-455. 4016141 10.1016/0005-2760(85)90114-6

[48] Kyburz D, Brentano F, Gay S Mode of action of hydroxychloroquine in RA-evidence of an inhibitory effect on toll-like receptor signaling. Nat Clin Pract Rheumatol. 2006;2(9):458-459. 16951696 10.1038/ncprheum0292

[49] Goldman FD, Gilman AL, Hollenback C, Kato RM, Premack BA, Rawlings DJ Hydroxychloroquine inhibits calcium signals in T cells: a new mechanism to explain its immunomodulatory properties. Blood. 2000;95(11):3460-3466. 10828029

[50] Lesiak A, Narbutt J, Sysa-Jedrzejowska A, Lukamowicz J, McCauliffe DP, Wozniacka A Effect of chloroquine phosphate treatment on serum MMP-9 and TIMP-1 levels in patients with systemic lupus erythematosus. Lupus. 2010;19(6):683-688. 20064914 10.1177/0961203309356455

[51] Golden EB, Cho HY, Hofman FM, Louie SG, Schonthal AH, Chen TC Quinoline-based antimalarial drugs: a novel class of autophagy inhibitors. Neurosurg Focus. 2015;38(3):E12. 10.3171/2014.12.FOCUS14748 25727221

[52] Pellegrini P, Strambi A, Zipoli C, et al Acidic extracellular pH neutralizes the autophagy-inhibiting activity of chloroquine: implications for cancer therapies. Autophagy. 2014;10(4):562-571. 24492472 10.4161/auto.27901 PMC3984580

